# Mucopolysaccharidosis VII in Brazil: natural history and clinical findings

**DOI:** 10.1186/s13023-021-01870-w

**Published:** 2021-05-22

**Authors:** Roberto Giugliani, Anneliese Lopes Barth, Melissa Rossi Calvão Dumas, José Francisco da Silva Franco, Liane de Rosso Giuliani, Carlos Henrique Paiva Grangeiro, Dafne Dain Gandelman Horovitz, Chong Ae Kim, Emilia Katiane Embiruçu de Araújo Leão, Paula Frassinetti Vasconcelos de Medeiros, Diego Santana Chaves Geraldo Miguel, Maria Espírito Santo Almeida Moreira, Helena Maria Guimarães Pimentel dos Santos, Luiz Carlos Santana da Silva, Luiz Roberto da Silva, Isabel Neves de Souza, Tatiele Nalin, Daniel Garcia

**Affiliations:** 1 Medical Genetics Service, HCPA, Department of Genetics, UFRGS, INAGEMP and DR Brasil Research Group, HCPA, Rua Ramiro Barcelos 2350, Porto Alegre, RS 90035-903 Brazil; 2 Medical Genetics Department, National Institute of Women, Children, and Adolescent Health, Oswaldo Cruz Foundation, Rio de Janeiro, RJ Brazil; 3 Rare Diseases Service - APAE Salvador, Salvador, BA Brazil; 4 Hospital Infantil Sabará -SP and University of São Paulo/ IPEN, São Paulo, SP Brazil; 5 Faculty of Medicine, Federal University of Mato Grosso Do Sul and Hospital Universitário Maria Aparecida Pedrossian (HUMAP/UFMS), Campo Grande, MS Brazil; 6 Complexo Hospitalar, Federal University of Ceará, Fortaleza, CE Brazil; 7 Instituto da Criança, Hospital das Clínicas da Faculdade de Medicina University of São Paulo, São Paulo, SP Brazil; 8 Medical Genetics Service, Hospital Universitário Prof. Edgard Santos, Salvador, BA Brazil; 9 Federal University of Campina Grande, Campina Grande, PB Brazil; 10 Escola Bahiana de Medicina E Saúde Pública, Salvador, BA Brazil; 11 Hospital Infantil Lucidio Portella, Teresina, PI Brazil; 12 Laboratory of Inborn Errors of Metabolism, Institute of Biological Sciences, Federal University of Pará, INAGEMP, Belém, PA Brazil; 13 Hospital de Clínicas, Federal University of Uberlândia, Uberlândia, MG Brazil; 14 Hospital Universitário Bettina Ferro de Souza, Federal University of Pará, Belém, PA Brazil; 15 Ultragenyx Brasil Farmacêutica Ltda, São Paulo, SP Brazil

**Keywords:** Mucopolysaccharidosis type VII, Sly disease, Lysosomal storage disorder, Glycosaminoglycans, Beta-glucuronidase deficiency

## Abstract

**Background:**

Mucopolysaccharidosis type VII (MPS VII), also known as Sly syndrome, caused by deficiency of the lysosomal enzyme β-glucuronidase, is an ultra-rare disorder with scarce epidemiological data and few publications about natural history and clinical spectrum.

**Methods:**

We conducted a case series report which included retrospective data from all MPS VII patients diagnosed through the “MPS Brazil Network” who were known to be alive in 2020 in Brazil (N = 13). Clinical data were obtained from a review of the medical records and descriptive statistics and variables were summarized using counts and percentages of the total population.

**Results:**

The majority of the patients were from the Northeast region of Brazil. Among the signs and symptoms that raised the clinical suspicion of MPS, coarse face was the most frequent; 58% of the patients had a history of non-immune hydrops fetalis. All the subjects presented short neck and trunk. The majority presented typical phenotypical signs of MPS disorders. They all presented neurodevelopmental delay and cognitive impairment. About half of this cohort had knees deformities. *Dysostosis multiplex* was identified in almost all patients and cardiomyopathy was less frequent than observed in other types of MPSs. The mean age at diagnosis was 5 years, ranging from 1 to 14 years. Almost all patients (12/13) were homozygous for the c.526C>T (p.Leu176Phe) mutation. A novel variant of the *GUSB* gene was found, the c.875T>C (p.Leu292Pro), in a compound heterozygous with the c.526C>T (p.Leu176Phe) variant.

**Conclusions:**

This case series is the biggest data collection of MPS VII patients alive in Latin America. The overall clinical picture of the MPS VII patients is very similar to other MPS disorders, including a spectrum of severity and delayed diagnosis.

## Background

Mucopolysaccharidosis VII (MPS VII), also known as Sly syndrome, is a heterogeneous, progressive, metabolic disorder caused by autosomal recessive inherited loss of function mutations at the *GUSB* gene (OMIM# 253220), leading to deficiency of the lysosomal enzyme β-glucuronidase (GUS: β-D-glucuronoside glucuronosohydrolase) [1–3] and consequent storage of glycosaminoglycans (GAGs).

The *GUSB* gene spans 20 kb on chromosome 7q11.21–7q11.22 and contains 12 exons; 56 disease-causing variants in *GUSB* (Beta Glucuronidase gene) have been reported to date in patients with MPS VII (Human Gene Mutation Database Professional). GUSB deficiency leads to systemic accumulation of the glycosaminoglycans (GAGs) chondroitin sulfate (CS), dermatan sulfate (DS), and heparan sulfate (HS) in the lysosomes of many tissues, causing multiple organ dysfunction and reduced life expectancy [4, 5].

MPS VII is an ultra-rare disorder with scarce epidemiological data and an estimated worldwide birth prevalence of less than 1:1,000,000 [6, 7]. Most patients with MPS VII have typical dysmorphism, short stature, skeletal dysplasia, hernias, hepatosplenomegaly, and a large proportion have cognitive impairment [4, 5]. The phenotype of MPS VII is highly heterogeneous, ranging from lethal non-immune hydrops fetalis (NIHF) to more attenuated late-onset phenotypes with survival to adulthood and normal or near-normal intelligence. The first case was reported in 1973 by Dr. William Sly [1]; since then, many other cases were identified in patients from countries of all continents. The first Brazilian cases with clinical, biochemical and molecular diagnosis of MPS VII were reported in 2003 in a family with three affected siblings [8].

A specific enzyme replacement therapy (ERT) was developed for MPS VII patients. Vestronidase alfa is a formulation of recombinant human β-glucuronidase (rhGUS; UX003—Ultragenyx Pharmaceutical Inc.), approved by the Food and Drug Administration (FDA) in 2017 and in 2018 by *Agência Nacional de Vigilância Sanitária* (ANVISA-Brazil) to treat children and adults with MPS VII. These approvals were based on findings from studies including non-interventional reviews of medical records, patient surveys, and clinical trials [9–13].

The extreme rarity of MPS VII means that there is very limited information available about natural history and clinical course. The aim of this article is to describe the clinical, biochemical and molecular findings of a case series of Brazilian MPS VII patients. This is, to the best of our knowledge, the largest cohort of living patients with MPS VII from a single country.

## Methods

This case series study included retrospective data from all Brazilian patients diagnosed with MPS VII through the “MPS Brazil Network” (“Rede MPS Brasil”) who were known to be alive and living in Brazil in 2020 [14]. Due to the lack of data of clinical data on patients deceased or lost to follow-up, they were not included in the present analysis. The MPS Brazil Network is a partnership involving Brazilian medical centers that treat MPS patients and the Medical Genetics Service of Hospital de Clínicas de Porto Alegre (MGS-HCPA). MGS-HCPA service coordinates the project, provides the necessary information and performs the biochemical and genetic analyses, results of which are forwarded to network members of participating centers in the whole country [15]. The ethical approval of this study is covered by the MPS Brazil Network project (#03-066/GPPG/HCPA) and all the patients or caregivers gave written informed consent to this publication.

The MPS Brazil Network has records of 13 living patients as of date, out of a total of 27 MPS VII Brazilian cases diagnosed from 1982 to 2019 (unpublished MPS Brazil Network data). All treating physicians were contacted and invited to a meeting to discuss the report and agreed to send available data of their patients. Patient clinical data were obtained from a review of the medical records using a standardized form developed by the study members.

Descriptive statistics was applied and variables were summarized using counts and percentages of the total population. Although all the information from participating centers was compiled, information on some items could not be obtained or were not available. Variables were summarized using descriptive statistics including mean, median, ranges, percentages and/or frequencies. Unless otherwise reported, the presented data refers to the latest assessment.

## Results

**Fig. 1:**
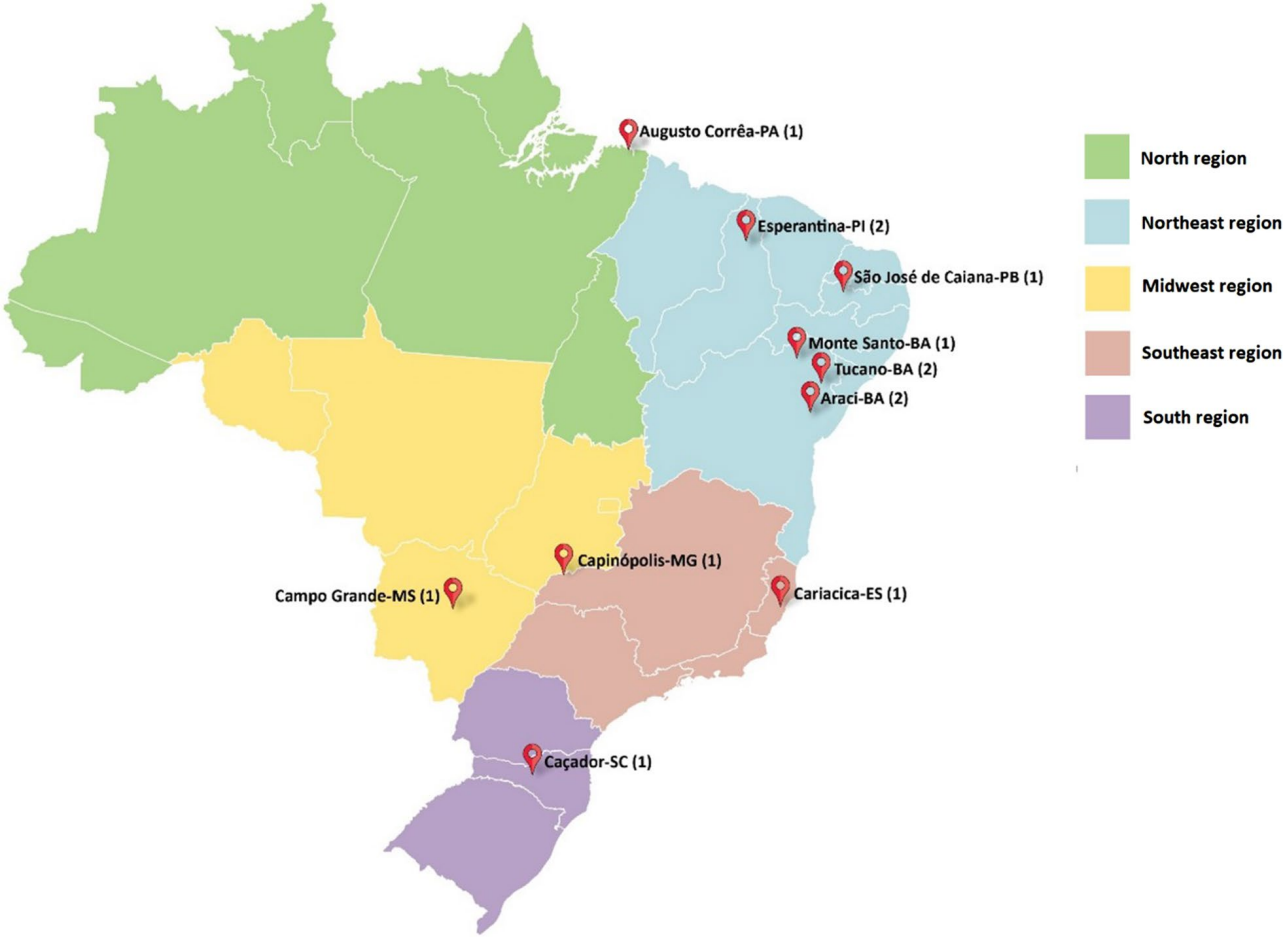
Geographical distribution of families with MPS VII (number of patients is in brackets)

This study collected data on 13 patients, 6 males and 7 females, from 11 unrelated families, ranging in age from 2 to 23 years (mean 12.9; median 13.5). The majority of the patients were from the Northeast region of Brazil (N = 8), two were from the Southeast region, one from the Center-West region, one from South region, and one from the North region (Fig. 1). The father of the patient from the Center-West region was originally from Pernambuco, a State in the Northeast region of the country. In seven families the parents of affected patients have some degree of consanguinity.

Among the signs and symptoms that raised the clinical suspicion of MPS, coarse face was the most frequent (3/10), followed by NIHF (2/10) and dysostosis multiplex (2/10). Short stature, hepatosplenomegaly or joint deformities was the leading diagnostic symptom for three patients. Parental consanguinity and family history were also observed by physicians as warning signs at the time of diagnosis. The information about the triggering factor for clinical diagnosis was not available for three patients. The mean age at diagnosis was 5 years, ranging from 1 to 14 years. Additional patient demographics, family history, and individual backgrounds are described in Table 1.

**Table 1:** Patient demographics, family and individual history

	Patient												
	1	2	3	4	5	6	7	8	9	10	11	12	13
Age (years)	1.5	2.5	3	9	9	10	13.5	13.5	19	21	21	22	23
Gender (F/M)	F	M	M	F	M	F	F	F	M	M	M	F	F
Parental consanguinity	Y	N	N	Y	Y	Y	Y	Y	Y	N	Y	N	Y
MPS VII positive family history	Y	N	N	N	Y	Y	N	N	Y	N	N	N	Y
NIHF	Y	Y	N	N	Y	Y	Y	N/A	N	Y	N	Y	N
Snoring	N	N/A	N	Y	N	N	Y	N/A	Y	?	Y	Y	N
Sleep apnea	N	N/A	N	N/A	Y	N	N	N/A	Y	?	Y	N	N
Recurrent respiratory infect	Y	N/A	N/A	Y	Y	Y	N	N/A	Y	Y	Y	Y	N
Hearing loss	N	N/A	N/A	N/A	Y	Y	N	NA	Y	Y	Y	N/A	N
GAGs excretion (mg/mMol Cr)	N/A	404	608,05	179	N/A	N/A	334	N/A	237	155	25	N/A	357
b-glucuronidase activity in leucocytes or plasma*	1.4	0.15	0.7#	1.7	0.11	0.37	0.8	0.2	N/D	N/D#	0.34	0.7	2.9#
Molecular results	HOM	HOM	C.HET	HOM	HOM	HOM	HOM	HOM	HOM	HOM	HOM	HOM	HOM
	c.526C>T (p.Leu176Phe)	c.526C>T (p.Leu176Phe)	c.526C>T (p.Leu176Phe)/c.875T>C (p.Leu292Pro)	c.526C>T (p.Leu176Phe)	c.526C>T (p.Leu176Phe)	c.526C>T (p.Leu176Phe)	c.526C>T (p.Leu176Phe)	c.526C>T (p.Leu176Phe)	c.526C>T (p.Leu176Phe)	c.526C>T (p.Leu176Phe)	c.526C>T (p.Leu176Phe)	c.526C>T (p.Leu176Phe)	c.526C>T (p.Leu176Phe)

N/A, not available; Y, yes; N, no; N/D not detected GUSB activity HOM, homozygous C.HET, compound Heterozygous; F, female; M, male; NIHF, non-immune Hydrops Fetalis

*Ref range, leucocytes 23–151 nMol/h/mg protein, plasma 30–300 nMol/h/mL

#b-glucuronidase activity in plasma

**Fig. 2:**
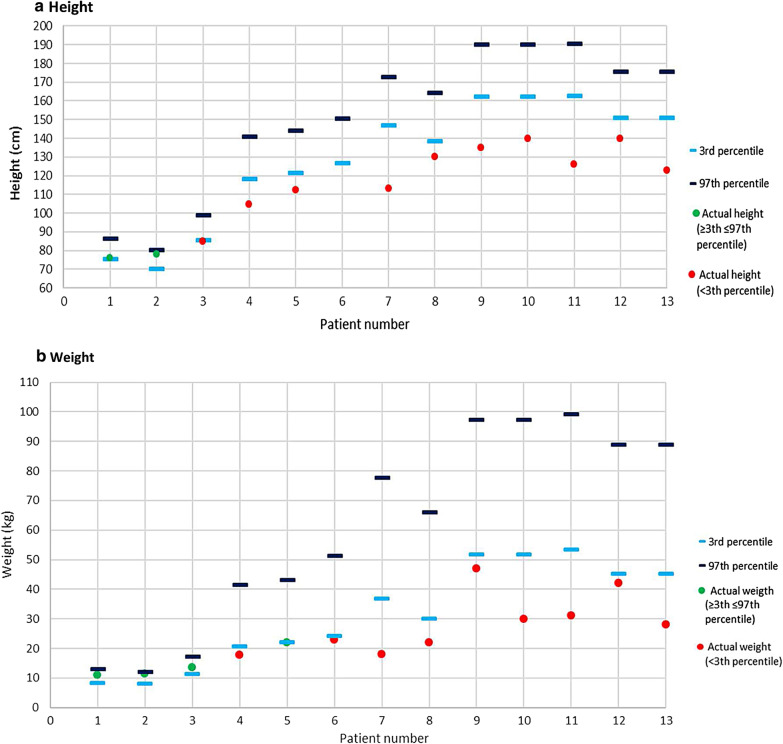
Anthropometric measures in the last assessment **a** height, **b** weight, with 3rd and 97th percentile obtained from WHO charts [16])

Anthropometric measures at the last assessment are described in Fig. 2. The 3rd and 97th percentile in the graphs were obtained from WHO (World Health Organization) charts [16] Patients numbered 7 and 12 both participated in a CLUX003-301 clinical trial (NCT02230566) and have been receiving ERT with alfa vestronidase since 2015 [12]. Therefore, the clinical course of their disease might be modified by the therapy during the period of the assessments here described.

Clinical exams at last assessment are described in Table 2. All the subjects presented short neck and trunk. The majority presented typical phenotypical signs, such as thick eyebrows, coarse facial features and abdominal protrusion. They all presented neurodevelopmental delay and cognitive impairment, while hyperactivity and seizures were less frequent. Four patients had *genu valgum*, three had *genu varum*, and three had no alterations on the knees. One patient had no foot alterations, four presented *equinus* feet (one of them slightly *valgum*), two *valgum*, three *varum*, and three unknown/not assessed. Most patients had dysostosis multiplex (11/13; 2 unknown); 10/13 did not have cardiomyopathy and the status of the remaining three were unknown/not assessed.

**Table 2:** Summary of clinical findings in the last assessment

Clinical data			Yes	Not	Unknown
Head and neck	Coarse facies		10	3	0
	Corneal clouding		8	5	0
	Thick eyebrows		10	3	0
	Macroglossia		9	4	0
	Dental changes		10	3	0
	Gum hypertrophy		9	3	1
	Short neck		13	0	0
Trunk	Short trunk		13	0	0
	Kyphosis		7	6	0
	Scoliosis		9	4	0
	Gibbosity		7	5	1
	Pectus carinatum/excavatum		9	4	0
	Abdominal protrusion		11	2	0
	Hernia		10	1	2
	Visceromegaly palpable	Liver	6	6	1
		Spleen	5	7	1
Limbs	Joint contractures		6	7	0
	Restricted mobility		10	3	0
	Claw hands		5	7	1
	Curved fingers		6	6	1
Neurological	Neuromotor development delay		13	0	0
	Cognitive deficit		11	1	1
	Limited vocabulary		12	0	1
	Behavior disturbances		3	9	1
	Seizures		1	11	1
Radiographic and complementary exams findings	Reduced pulmonary function (CVF)		0	5	7
	Valvular heart disease		7	3	3
	Cardiomyopathy		0	11	2
	Dysostosis multiplex		13	0	0
	Acetabular dysplasia in the hips		9	2	2
	Hepatosplenomegaly		6	5	2

## Discussion

MPS VII is an ultra-rare genetic disorder, one of the least prevalent MPS types. This case series is the biggest MPS VII data collection of patients alive in Latin America, and as such, contributes to a better understanding of this highly heterogeneous condition. In Brazil, during the last 40 years, 27 MPS VII patients were diagnosed by MGS-HCPA and MPS Brazil Network, representing 1.3% of the total MPS cases (unpublished data of the MPS Brazil Network). The 13 patients described in this case series include all MPS VII patients known to be alive in the country. This is likely representative of the actual number and geographical distribution of MPS VII patients in Brazil, as MGS-HCPA is the main reference center for the diagnosis of MPS disorders in the country, receiving samples from every Brazilian state since it was established in 1982 [15, 17].

In Brazil, the overall mean rate of consanguineous marriages is about 4.8%, the lowest inbreeding levels were described in the southern states, whereas in some states of the Northeast these rates vary from 6 to 12% [18, 19]. This could potentially explain why most of the MPS VII patients described here are from this region. Nine out of the 13 patients described here have history of parental consanguinity, seven of which are from the Northeast region.

As previously described in MPS VII [20–22], patients can present a history of NIHF. In our study 58% (7/12) of the patients had a history of NIHF, a proportion higher even than that found in previous case series, with percentages reported as 38.5% [22], 41.1% [4], 45.5% [23] and 48.6% [20], which reinforces the importance of testing for MPS VII during the etiologic investigation of an hydropic fetus.

As in the cohort described by Montano et al. [4], some phenotypic characteristics are frequently reported in patients with MPS VII and should lead to a suspicion of MPS, highlighting coarse face, thick eyebrows, short trunk and short neck. Montano further described that dysostosis multiplex on X-ray was the most consistent finding in the MPS VII patient survey (90% of the patients) [4], which is in accordance with our findings, as 100% of our patients presented this abnormality.

Cognitive impairment is common in MPS VII patients and has been attributed to the storage of the GAG heparan sulfate (HS). This finding is present in 86 to 94% of patients, according to Montano. Since MPS VII is a progressive disease, due to continuous accumulation of GAGs, patients usually deteriorate cognitively during the course of the disease [24]. All Brazilian patients described here have neuromotor development, cognitive, and/or behavioral disturbances.

The skeletal phenotype of the patients described here is very typical of MPS, with patients presenting a set of abnormalities known as “dysostosis multiplex”. However, some characteristics seem to be more prominent in MPS VII patients, such as very short trunk and an apparent proportionally long lower extremities with feet deformities. One of the patients with congenital talipes equinovarus was due to neuro tube defect.

Short stature is one of the most characteristic features observed in patients with MPS VII. However, in this survey, it was not observed in patients under three years of age. Previous cohort studies have also reported normal growth until 18–24 months of age [4]. These findings suggest that short stature is not a hallmark for early MPS VII diagnosis.

Regarding the organomegaly often found in MPS, 50% of the patients from this cohort have hepatomegaly/splenomegaly, compared to 75% from Montano et al. [4], 39.8% from Zielonka et al. [23] and 46.2% from Morrison et al. [22]. The fact that 2 out of 13 patients were receiving ERT may have an impact on the proportion of hepatomegaly/splenomegaly observed in our cases. Umbilical and/or inguinal hernia was found in 91% while in other cases ranged from 31.8 to 61% [4, 22, 23].

The cardiorespiratory impairment, which is an important cause of morbidity in patients with MPS, was not so evident in our cohort as from other reports [4, 22]. This may not reflect that the patients in Brazil are less severe, but simply that cardiac and pulmonary assessments were available for only a few patients.

Regarding the 14 patients diagnosed by MGS-HCPA and MPS Brazil Network since 1982 but not included in this analysis, we have the following comments: 1)one patient moved to the United States shortly after receiving the diagnosis and we know is alive and receiving ERT;2) six patients we know that have passed away; of the six deceased patients, three were siblings of a consanguineous couple, described by Schwartz et al. (2003) as the first report of MPS VII in a Brazilian family: one died at 6 years from respiratory problems, another died at 20 months due to complications of bone marrow transplantation and the patient who was 18 years old at the time of publication, died later. The three siblings had early onset of symptoms, dysostosis multiplex and did not have NIHF [8]. Another deceased patient was a girl from the city of Araci, in Bahia, which is in the same micro-region of five patients reported here (cities of Monte Santo, Tucano and Araci, Fig. 1). This patient had a similar phenotype, including NIHF, dysostosis multiplex, hepatomegaly and behavioral problems; she died of pneumonia at age 12. We are aware that two other patients died, but we do not have clinical details; 3) the remaining seven patients were diagnosed but no clinical details are available and no follow-up was possible.

One peculiar characteristic of the Brazilian cohort is that almost all patients are homozygous for the c.526C>T (p.Leu176Phe) mutation. This mutation was first identified in a Mennonite family and was the fourth mutation in *GUSB* gene reported [25]. This is the most common mutation among MPS VII patients worldwide, with an overall allele frequency of 20.4% [26]. The phenotype-genotype correlation for this mutation is still not clear as some patients were described as moderately severe [8, 25] while others as presenting an attenuated phenotype [26]. We also observed a broad clinical spectrum for this genotype in our study, with a tendency to have patients at the severe end of the spectrum. This level of severity might not be expected based on data from biochemical assays and mouse models, which suggest that the c.526C>T (p.Leu176Phe) GUS allele has 84% of the enzyme activity of wild-type GUS [26, 27].

One patient in this cohort is a compound heterozygote for c.526C>T (p.Leu176Phe) and c.875T>C (p.Leu292Pro), a mutation was not previously reported. This patient has a moderate phenotype despite presenting with very low GUS plasma activity (0.7 nMol/h/mg). In fact, all 13 patients in this study had very low enzyme activity, which does not seem to be predictive of their clinical phenotypes. The heterozygous patient presented initially with persistent jaundice, hepatosplenomegaly and umbilical hernia and by the age of 3, presented coarse facial features, neurodevelopmental delay, and skeletal dysplasia.

Both patients receiving ERT could be considered with a moderate/severe phenotype, they had history of NIHF and all typical MPS VII clinical features, including neurocognitive impairment. Although the objective of this study was not to evaluate the effects of the ERT, it becomes clear that some signs as hepatosplenomegaly were reduced and the progression of the disease was apparently stabilized.

## Conclusions

The overall clinical picture of the MPS VII patients is very similar to other MPS disorders, including a broad spectrum of severity, as observed in the Brazilian patients. This spectrum of severity is characterized by patients who died in the first years of life, from complications of the disease, to patients who reached the third decade of life, with the disease relatively stable, even before the availability of specific treatments. This clinical heterogeneity is somewhat surprising considering that all the living patients but one are homozygous for the same missense mutation c.526C>T(p.Leu176Phe). Therefore, it seems very challenging to establish whether there is a genotype–phenotype correlation for this ultra-rare condition. The novel variant c.875T>C (p.Leu292Pro), identified in heterozygosity in one patient, is being described here for the first time.

The geographical origin of the patients described here matches with regions in Brazil with high consanguinity rates, which is in line with the occurrence of cases of ultra-rare disorders. The fact that patients from different families with the same homozygous mutation were identified in different regions of the country suggests that the mutation arrived in Brazil long ago. Molecular genetic studies on the ancestrality of this mutation are planned to provide further trace its path in Brazil.

It is also important to reinforce the need to consider MPS VII as a differential diagnosis for NIHF, as most patients described in our series had a history of fetal hydrops. In fact, the majority of patients presented here had a delayed diagnosis. With the availability of a targeted treatment, early diagnosis and treatment is key to improve clinical outcomes, as seen for other MPS that also have specific treatment.

## Data Availability

The datasets are not publicly available due to individual privacy reasons (patients’ confidentiality).

## Abbreviations

ANVISA: National Health Surveillance Agency (*Agência Nacional de Vigilância Sanitária*)
CS: Chondroitin sulfate
DS: Dermatan sulfate
ERT: Enzyme replacement therapy
FDA: Food and Drug Administration
GAGs: Glycosaminoglycans
GUS: β-D-glucuronoside glucuronosohydrolase
GUSB: Beta Glucuronidase
HS: Heparan sulfate
MGS-HCPA: Medical Genetics Service of Hospital de Clínicas de Porto Alegre
MPS: Mucopolysaccharidosis
MPS VII: Mucopolysaccharidosis VII
NIHF: Non-immune hydrops fetalis
WHO: World Health Organization

## References

[1] Sly WS, Quinton BA, McAlister WH, Rimoin DL (1973). Beta glucuronidase deficiency: report of clinical, radiologic, and biochemical features of a new mucopolysaccharidosis. J Pediatr.

[2] Neufeld EF, Muenzer J, Valle DL, Antonarakis S, Ballabio A, Beaudet AL, Mitchell GA (2007). The mucopolysaccharidoses. (2007) Online metabolic and molecular bases of inherited disease.

[3] OMIN -Online Mendelian Inheritance in Man. MUCOPOLYSACCHARIDOSIS, TYPE VII; MPS7 [Internet]. # 253220. 2020 [cited 2020 Aug 26]. https://omim.org/entry/253220.

[4] Montaño AM, Lock-Hock N, Steiner RD, Graham BH, Szlago M, Greenstein R (2016). Clinical course of sly syndrome (mucopolysaccharidosis type VII). J Med Genet BMJ Publishing Group.

[5] Muenzer J (2011). Overview of the mucopolysaccharidoses. Rheumatology.

[6] Orphanet. Mucopolysaccharidosis type 7 [Internet]. portal rare Dis. orphan drugs. 2019 [cited 2020 Aug 26]. https://www.orpha.net/consor/cgi-bin/OC_Exp.php?lng=en&Expert=584.

[7] Federhen A, Pasqualim G, Freitas TF, Gonzalez EA, Trapp F, Matte U (2020). Estimated birth prevalence of mucopolysaccharidoses in Brazil. Am J Med Genet Part A.

[8] Schwartz I, Silva LR, Leistner S, Todeschini LA, Burin MG, Pina-Neto JM (2003). Mucopolysaccharidosis VII: clinical, biochemical and molecular investigation of a Brazilian family. Clin Genet.

[9] McCafferty EH, Scott LJ (2019). Vestronidase alfa: a review in mucopolysaccharidosis VII. BioDrugs.

[10] Fox JE, Volpe L, Bullaro J, Kakkis ED, Sly WS (2015). First human treatment with investigational rhGUS enzyme replacement therapy in an advanced stage MPS VII patient. Mol Genet Metab.

[11] Jones SA, Ghosh A, Breen C, Kakkis ED, Sly WS (2015). Enzyme replacement therapy (ERT) for mucopolysaccharidosis VII (MPS VII; Sly syndrome) reduces lysosomal storage in a 36-week phase 1/2 clinical study. Mol Genet Metab.

[12] Harmatz P, Whitley CB, Wang RY, Bauer M, Song W, Haller C (2018). A novel Blind Start study design to investigate vestronidase alfa for mucopolysaccharidosis VII, an ultra-rare genetic disease. Mol Genet Metab.

[13] Wang RY, da Silva Franco JF, López-Valdez J, Martins E, Sutton VR, Whitley CB (2020). The long-term safety and efficacy of vestronidase alfa, rhGUS enzyme replacement therapy, in subjects with mucopolysaccharidosis VII. Mol Genet Metab.

[14] Rede MPS Brasil [Internet]. [cited 2020 Mar 27]. http://www.ufrgs.br/redempsbrasil/sobremps.php.

[15] Giugliani R, Vairo FP, Riegel M, De Souza CFM, Schwartz IVD, Pena SDJ (2016). Rare disease landscape in Brazil: report of a successful experience in inborn errors of metabolism Dr Segolene Ayme. Orphanet J Rare Dis.

[16] World Health Organization. WHO Chart catalogue [Internet]. 2021 [cited 2021 Mar 4]. https://www.who.int/childgrowth/standards/chart_catalogue/en/.

[17] Vieira TA, Trapp FB, De Souza CFM, Faccini LS, Jardim LB, Schwartz IVD (2019). Information and diagnosis networks—tools to improve diagnosis and treatment for patients with rare genetic diseases. Genet Mol Biol Braz J Genet.

[18] Freire-Maia N (1990). Genetic effects in Brazilian populations due to consanguineous marriages. Am J Med Genet Am J Med Genet.

[19] Santos S, Kok F, Weller M, de Paiva FRL, Otto PA (2010). Inbreeding levels in Northeast Brazil: Strategies for the prospecting of new genetic disorders. Genet Mol Biol Braz J Genet.

[20] Holtz M, Montaño AM, Sly WS (2020). Association between mucopolysaccharidosis Type VII and hydrops fetalis. Ultrasound Obstet Gynecol.

[21] Cheng Y, Verp MS, Knutel T, Hibbard JU (2003). Mucopolysaccharidosis type VII as a cause of recurrent non-immune hydrops fetalis. J Perinat Med.

[22] Morrison A, Oussoren E, Friedel T, Cruz J, Yilmaz N (2019). Pathway to diagnosis and burden of illness in mucopolysaccharidosis type VII-A European caregiver survey. Orphanet J Rare Dis.

[23] Zielonka M, Garbade SF, Kölker S, Hoffmann GF, Ries M (2017). Quantitative clinical characteristics of 53 patients with MPS VII: a cross-sectional analysis. Genet Med.

[24] Wegrzyn G, Jakóbkiewicz-Banecka J, Narajczyk M, Wiśniewski A, Piotrowska E, Gabig-Cimińska M (2010). Why are behaviors of children suffering from various neuronopathic types of mucopolysaccharidoses different?. Med Hypotheses Med Hypotheses.

[25] Wu BM, Tomatsu S, Fukuda S, Sukegawa K, Orii T, Sly WS (1994). Overexpression rescues the mutant phenotype of L176F mutation causing beta-glucuronidase deficiency mucopolysaccharidosis in two Mennonite siblings. J Biol Chem United States.

[26] Tomatsu S, Montano AM, Dung VC, Grubb JH, Sly WS (2009). Mutations and polymorphisms in GUSB gene in mucopolysaccharidosis VII (sly syndrome). Hum Mutat.

[27] Tomatsu S, Orii KO, Vogler C, Grubb JH, Snella EM, Gutierrez MA (2002). Missense models [Gustm(E536A)Sly, Gustm(E536Q)Sly, and Gustm(L175F)Sly] of murine mucopolysaccharidosis type VII produced by targeted mutagenesis. Proc Natl Acad Sci U S A.

